# Study on the Surface of Cobalt-Chromium Dental Alloys and Their Behavior in Oral Cavity as Cast Materials

**DOI:** 10.3390/ma15093052

**Published:** 2022-04-22

**Authors:** Willi Andrei Uriciuc, Adina Bianca Boșca, Anida-Maria Băbțan, Horațiu Vermeșan, Cecilia Cristea, Mihaela Tertiș, Petru Pășcuță, Gheorghe Borodi, Maria Suciu, Lucian Barbu-Tudoran, Cătălin Ovidiu Popa, Aranka Ilea

**Affiliations:** 1 Faculty of Dental Medicine, “Iuliu Hațieganu” University of Medicine and Pharmacy, 400012 Cluj-Napoca, Romania; willi.uriciuc@umfcluj.ro (W.A.U.); babtan.anida@umfcluj.ro (A.-M.B.); aranka.ilea@umfcluj.ro (A.I.); 2 Faculty of Medicine, “Iuliu Hațieganu” University of Medicine and Pharmacy, 400012 Cluj-Napoca, Romania; 3 Faculty of Materials and Environmental Engineering, Technical University of Cluj-Napoca, 400641 Cluj-Napoca, Romania; horatiu.vermesan@imadd.utcluj.ro (H.V.); petru.pascuta@phys.utcluj.ro (P.P.); catlin.popa@stm.utcluj.ro (C.O.P.); 4 Faculty of Phrmacy, “Iuliu Hațieganu” University of Medicine and Pharmacy, 400349 Cluj-Napoca, Romania; ccristea@umfcluj.ro (C.C.); mihaela.tertis@umfcluj.ro (M.T.); 5 National Institute for R&D of Isotopic and Molecular Technologies (INCEDETIM), 400293 Cluj-Napoca, Romania; borodi@itim-cj.ro; 6 LIME-CETATEA, National Institute for R&D of Isotopic and Molecular Technologies (INCEDETIM), 400293 Cluj-Napoca, Romania; suciuc.maria@ubbcluj.ro (M.S.); lucian.barbu@ubbcluj.ro (L.B.-T.); 7 Electron Microscopy Center, Biology and Geology Faculty, Babes-Bolyai University, Clinicilor 5-7, 400007 Cluj-Napoca, Romania

**Keywords:** cobalt–chromium dental alloys, casting, corrosion, SEM, EDX, XRD, oxidation

## Abstract

This study presents the correct processing of Co–Cr alloys as a method of preserving the properties of the materials as-cast, and therefore they can be safely placed in contact with the oral cavity tissues as resistance frameworks. The basic materials analyzed in this study were five commercial Co–Cr dental alloys with different components obtained in three processing steps. The analysis of the electrochemical behavior at the surface of the Co–Cr alloys was performed by electrochemical measurements: impedance spectroscopy (EIS), open circuit electrical potential (OCP), and linear polarization (LP). In terms of validation, all five alloys had a tendency to generate a stable oxide layer at the surface. After the measurements and the graphical representation, the alloy that had a higher percentage of tungsten (W) and iron (Fe) in composition showed a higher tendency of anodizing. After the application of the heat treatment, the disappearance of the hexagonal phase was observed, with the appearance of new phases of type (A,B)_2_O_3_ corresponding to some oxide compounds, such as Fe_2_O_3_, Cr_2_O_3_, (Cr,Fe)_2_O_3_, and CoMnO_3_. In conclusion, the processing of Co–Cr alloys by melting and casting in refractory molds remains a viable method that can support innovation, in the context of technology advance in recent years towards digitalization of the manufacturing process, i.e., the construction of prosthetic frameworks conducted by additive methods using Co–Cr powder alloy.

## 1. Introduction

In dentistry, oral rehabilitation treatments are performed using prosthetic works that restore the masticatory function, and, from a constructive point of view, their stability must be ensured by resistance frameworks, which are currently made of metallic materials, such as Cobalt–Chromium (Co–Cr) alloys [1]. Dental restorations represent just a part of biomaterial applications in dentistry when adhesion aspects and surface modifications strongly influence the functionality of medical devices [2].

Metallic materials are considered materials of choice in the manufacture of prosthetic frameworks. Co–Cr alloys, or stellites, are commonly used in dental prosthetics [3]. There are different types of metal corrosion: inter-granular, pitting, crevice, fatigue, stress, fretting, and galvanic. On the other hand, in many cases (such as metal-on-metal prosthesis) corrosion and wear appears between two metallic components [4]. The accessibility of the classical method of processing Co–Cr alloys by melting and casting gives these alloys a wide range of uses [5].

The method of manufacturing prosthetic frameworks by melting and casting Co–Cr alloys depends on the refractory shape and its deformation after heat treatment. Regardless of whether the model of the complementary structure was made on a duplicate refractory model or directly on the assembly surface, the method is influenced by the refractory material [6]. Although the refractory packing mass is specific to the molded alloy, in terms of the expansion on heating and the shrinkage on cooling of the materials, the complementary frameworks made by this method require adaptation by mechanical processes at the surfaces [7].

In order to perform a better dental application by casting, the cobalt–chromium alloy must have a homogenous and dendritic structure, without inclusions, which is possible when is used casting technology in protective controlled environment [8].

### 1.1. Corrosion at the Surface of Co–Cr Alloys

The biocompatibility of dental alloys is a critical issue, because the alloys remain in long-term intimate contact with tissues and saliva [9] on the oral cavity that represents the prosthetic field [10,11]. To ensure biological safety [12], the most important property of alloys is resistance to corrosion in the oral cavity [13], where the alloys are exposed to a variation of pH, influenced by the nature of food and dental plaque. A low pH increases the release of metallic elements, acting as a corrosive medium [14].

The corrosion resistance of the surface of a metal prosthetic structure is the most important feature in the evaluation of the biocompatibility of the alloy [15]. The majority of stellites used in dentistry contain about 60% Co and 25% Cr, which ensure the biocompatibility of the alloy. Alloys may also contain small amounts of molybdenum (Mo), tungsten (W), and other metals [10,16].

Cobalt and chromium form a solid solution if the latter is found up to about 30%, according to the Co–Cr diagram. A value of 30% represents the solubility limit of Cr in Co. Exceeding this value makes the additional percentage of Cr to be found in the σ phase, in which it is extremely fragile, imprinting this feature on the entire alloy. For higher percentages of Cr, higher corrosion resistance is reached by these alloys [14,15,16].

Cr is the main alloying element, responsible for imprinting the passive character of the alloy. Added in a proportion of 11–33%, Cr increases the chemical stability of the alloy by forming a protective surface of oxide films. Cr improves resistance to oxidation at high temperatures, thus protecting the alloy against corrosion [15].

Cr forms oxide (Cr_2_O_3_) films, which are stable and adherent with a protective property, ensuring the minimization of the diffusion speed of metal ions at the surface. The resistance of alloys to chemical agents that initiate corrosion is due to the ability of Cr to form an adhesive and insoluble film, acting as a shield for the metal substrate [17].

The surface topography of the parts is marked by the method used at this level: mechanical processing (PM), sandblasting (S), oxidation by heat treatment (TTO), mechanical polishing (LM), or electrochemical (LE). Each of these surface conditioning processes influences the corrosion resistance of the part [18].

There are various methods of processing alloys for the manufacture of prosthetic structural components. Thus, we can mention the classic and established method of melting and casting alloy ingots in a refractory form obtained by melting the construction wax [19].

The accessibility of the melting and casting processing method provides these materials with a wide range of uses. The durability of prosthetic frameworks not only represents the ability of the material to retain its properties in the functional context, but also the ability of the environment in which they were designed to be used as resistance prosthetic frameworks [20] and as supports for aesthetic ceramic components that can be built on as the alloy–ceramic system in which chemical adhesion (after sintering) of the ceramic layer on the metal component is very important [21].

This manufacturing method influences the mechanical properties of the alloy and its microstructure after the fabrication of the framework [5].

### 1.2. Structure of Co–Cr Alloys

Co–Cr–Mo alloys cast by the conventional method are composed of two phases (phase ɣ, with a cubic structure with centred faces, and phase ɛ, with a hexagonal structure), being stable at both high and low temperatures. In order to improve the mechanical properties of Co–Cr–Mo alloys, heat treatments can be applied to the manufactured frameworks.

Applying a heat treatment at a high temperature of about 1100 °C can determine a good homogenization of the cast structure of dental cobalt alloys. Additionally, the dendrites become finer [22]. Following the heat treatment, the volume proportion of the two phases can vary, producing a homogeneous structure with improved mechanical properties [5,23,24,25].

Co–Cr–W as-cast alloys have a balanced biphasic structure ɣ + ɛ due to their composition, which differs proportionally from the two phases of Co–Cr–Mo alloys, whose random atomic arrangement has commonly happened in the as-cast product. This will always be a discussion. Studies have shown that, for the homogenization thermal treatment process, there is a transformation from a random (disordered) to a more ordered structure. The solution treatment method can also produce ɣ-Co phase (FCC) followed by the hexagonal structure as a minor phase [26].

The as-cast specimens only exhibit diffraction peaks assigned to the ɣ phase. In contrast, both c and e reflections are observed for the heat-treated specimens. Thus, the e phase forms as a result of an athermal martensitic transformation that occurs during cooling after the heat treatment [26,27].

The presence of the gamma phase can be induced in another way: by reaching the temperature at which phase ɛ exits in the stability zone, isothermally, by reaching a temperature between 650–950 °C as a result of bending the deformation favouring the martensitic transformation of the alloy [27].

Depending on the nature and chemical composition of the samples, the morphology is different. The microstructure of Co–Cr dental alloys depends on the manufacturing technique. Given the differences in microstructural properties among the tested specimens, further differences in their technological achievement and clinical behavior can be anticipated [28].

### 1.3. Oxidation of Co–Cr Alloys

Oxidation at the surface of prosthetic framework by heat treatment is a method used to improve the bonds between Co–Cr alloys and plating ceramics, resulting in a physiognomic prosthetic reconstruction [29].

In order to create an optimal layer of oxides but also to achieve an efficient decontamination on the surface of the structure, the temperature of the oxidative heat treatment (OTT) must be between 960 °C and 980 °C, in accordance with the instructions of the alloy manufacturer [30].

The characteristics of the oxide layer to be considered are: color, thickness, and adhesion to the alloy that produced it. The finishing processes of the metal part, such as sandblasting, acid etching, and hardening agents, influence the connection between the alloy and the cladding ceramics [31].

Mo reduces the intensity of the oxidation phenomenon, ensuring the passivation and decrease in the tendency of breaking of the passive film [32].

The oxide layers on the surface of Co–Cr–W alloys are structured as follows: an outer layer of CoO, an intermediate layer rich in Cr, and a deep layer rich in W. The rapid oxidation of Co–Cr–W alloys at high temperatures results in the formation of W oxide, which makes the oxide layer much more adherent and resistant [33].

Oxidation is a self-protection ability of the material that is active from the design stage. The oxide layers on the surface of Co–Cr base alloys can be considered as an elastic buffer [34].

## 2. Materials and Methods

The basic materials analyzed in this study were five commercial Co–Cr dental alloys. The alloys used were different, both in terms of the mass percentage of each main alloying element (Co, Cr) and in terms of the secondary alloying elements (Mo, W, Fe, N, Mn, Si).

### 2.1. Materials

The alloys were in the form of cylinders and could be identified under the trade name, and they were named in the study according to the W content of the alloy:

- Co59.5Cr31.5Mo5Si2Mn1.0 Super C (Dental Alloy Products) [35] alloy named: 0.
- Co61Cr26Mo6W5Fe0.5Si1 Wirobond C alloy (Bego Gmbh) [36] alloy named: 5.
- Co59Cr25Mo4W10Si1Mn0.8 alloy Heraenium P (Heraeus Kulzer) [37] alloy named: 10.
- Co55.2Cr24W15Fe4Si1Mn0.8 alloy Heraenium Pw (Heraeus Kulzer) [38] alloy named: 15.
- Co54.1Cr20W16.4Fe7.5Si1.5Mn0.3 Starloy Soft (Dentsply) [39] alloy named: 16.4.

Based on the alloys introduced in the study in the form of raw materials, the main groups of samples were made: start samples (A), cast samples (P), and oxidized samples (O).

The A samples (0-A, 5-A, 10-A, 15-A, and 16.4-A) were obtained by sectioning the Co–Cr alloy cylinders in the horizontal plane. The sectioning was performed using a rotary instrumented silicon carbide (SiC) disc (15 × 10^3^ rpm) by mounting on a dental micromotor (Forte 200α, Saeshin Precision Co., Ltd., Daegu, Korea) (see Figure S1). Previously, the surfaces of the A samples were mechanically machined (PM) with rotary, abrasive tools (Figure S2a), rubber (Figure S2b), and fibers (cotton wool) (Figure S2c) to obtain a mirror texture. The P samples went through the technical stages of a born-out model, made based on a multiplier silicone mold. The models were made of 100% organic modeling wax (High-precision modeling wax, Smile Line, Switzerland) using an electric knife (Waxlectric light II, Renfert Gmbh Co., Hilzingen, Germany) (Figure S3).

The packaging of the P and Pa models was made in phosphate-based refractory table paste (Bellavest SH/Begosol HE, BEGO Goldschlagerei Gmbh, Bremen, Germany) 160 g/40 mL soil (HE/H_2_O). The mixture was made using a vacuum mixer for 60 s and 350 rpm under vacuum 10–1.

For the heat treatment, a preheating furnace (P30-UGIN DENTAIRE, Seyssinet-Pariset, France, Seyssinet-Pariset, France) was used, in order to achieve the refractory form. The packaging was kept in the oven at 900 °C and for 30 min.

The melting and casting of the shaped alloy was performed using a melting machine with high frequency induction and centrifugal casting (Fornax T-BEGO Goldschlagerei Gmbh, Bremen, Germany) (Figure S4).

After casting, the alloy samples were allowed to cool in the mold until thermal equilibrium with the environment was reached. After cooling, samples P and Pa were mechanically extracted from the mold and cleaned by blasting with Al_2_O_3_ particles (175 μm granulation) under a pressure of 3.5 bar.

The surfaces of the P samples were mechanically processed (PM), as well as the surfaces of the A samples, with rotary, abrasive tools, gums, and fibers (cotton wool) until obtaining a mirror texture.

After the analysis, the P (G2) samples were subjected to oxidative heat treatment performed in three successive steps using a programmable heating furnace (Programmed P300, Ivoclar Vivadent, Schaan, Liechtenstein) (Figure S5).

### 2.2. Methods

#### 2.2.1. Corrosion

P-type samples were analyzed taking electrochemical behavior into consideration.

The experimental stands designed and adapted in this work were connected to a laboratory potentiostat (PGZ 100 VOLTALAB). The processing and interpretation of data were possible using the Voltamaster 4 software.

The electrochemical cell was composed of: the working electrode, represented by the surface of the alloy that was under investigation (Figure S6), the reference electrode type Ag/AgCl (3M KCl), and the counter electrode, or the auxiliary electrode, a Pt wire, and the electrolyte −20 mL Ringer’s solution (8.6 g NaCl, 0.33 g CaCl_2_, 0.3 g KCl and the rest H_2_O). The electrolytic cell was connected to the potentiostat using connectors to apply the frequency signal and record the impedance response of the sample. The working electrode was represented by an alloy surface consisting of P-type samples marked as follows: 0-P, 5-P, 10-P, 15-P, and 16.4-P, depending on their composition. To ensure a constant test surface, the working electrode was sealed with wax at the surface boundary to clearly delimit the investigation area (Figure S6). Moreover, the analyzed part was glued with wax to the cell containing the electrolyte (Figure S6).

The reference electrode and the counter electrode were positioned on a glass stand with a size adapted to the electrochemical cell and were connected to the potentiostat (Figure S7).

After arranging the electrochemical cell, the general settings of the potentiostat were made, as well as the individual settings, related to the working electrode.

##### Electrochemical Impedance Spectroscopy (EIS)

Electrochemical impedance spectroscopy (EIS) is a very well-known and frequently used method for the quantitative evaluation of the anticorrosive performance of protective coatings for different materials. This electrochemical method is also frequently used for studies on the corrosion resistance of some materials in the environmental conditions in which they are to be used. The advantage is that, after some tests, reliable data are obtained, which allows predicting the long-term performance of coatings as well as the maximum service life of those materials under operating conditions. The result of the EIS tests is the impedance spectrum of the electrochemical system, which can have several forms of representation, all of which are correlated with the variation of the system impedance with the applied frequency.

In practice, different types of EIS measurements can be performed to assess the corrosion resistance of a material or the degree of corrosion protection obtained from a coating.

The first type of test concerns the measurement of the performance of the material and the absorption of water during contact with the environment. This type of test provides relevant results within two to three weeks, but it can also be extended to long-term testing.

##### Open Circuit Potential

Linear polarization resistance is another rapid electrochemical method for measuring corrosion resistance. In experiments of this kind, the potential is swept in a small interval above and below its corrosion potential, and the resulting current of the system is measured. The polarization resistance (Rp), defined as the slope of the current–potential curve, is then evaluated from experimental data. 

##### Linear Polarization Measurement

The density of the corrosion current expressed in the loss of the thickness of the alloy over time was determined by the Tafel equation. From the experimental data, using the Volmer equation, the thermodynamic parameters βa and βc were calculated. The tests focused on applying a direct current potential to the surface of the analyzed sample and obtaining feedback.

Pitting corrosion is an extremely localized form of attack that results in the formation of holes (crevices) at the surface of the material. This type of corrosion is due to the localized destruction of the protection of the passive layer that covers the alloy, thus causing cracks, and it is usually encountered in aluminum and stainless steel of lower quality.

Since this is “pitting” corrosion and not general corrosion, it would not be correct to analyze the corrosion rate (V_cor_), but the value of the corrosion current density still can be taken into consideration (I_cor_).

#### 2.2.2. X-ray Diffractometry

The analysis on the surfaces of samples A, P, and O was performed by X-ray diffraction using the XRD-6000 Shimadzu Diffractometer, and X-ray diffractograms were obtained at room temperature, using a graphite monochromator for a Cu anode tube (λ = 1.540560).

#### 2.2.3. SEM—Scanning Electron Microscopy

P samples were analyzed by scanning electron microscopy (SEM) and EDS analysis, using a Hitachi SU8230 microscope, 30 kV, 10 μA, and 8 mm working distance (Hitachi Ltd., Tokyo, Japan), equipped with an EDX spectrometer.

## 3. Results

The heat treatment of the P (G2) samples was performed simultaneously in three cycles:

- OTI1: vacuum treatment with high temperature oxidation of samples P (G2), followed by samples O1 (5-O1, 15-O1).
- OTI2: high temperature oxidation vacuum heat treatment of O1 samples, followed by O2 samples (5-O2, 15-O2).
- OC3: heat treatment in atmospheric air with continuous oxidation of O2 samples, followed by O3 samples (5-O3, 15-O3).

**Figure 1:**
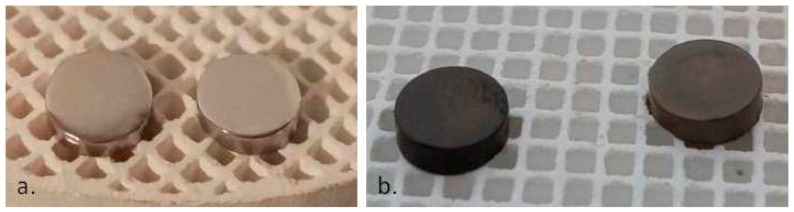
(**a**) Mechanically processed samples to the mirror texture; (**b**) Samples after application of oxidative heat treatment (5-O3 and 15-O).

The remaining P samples in the study,(5-P, 15-P), were subjected to the same heat treatment, which at the end of the treatment resulted samples 5-O3 and 15-O3, respectively (Figure 1).

### 3.1. Corrosion

The ability to form a passive protective film on the surface of the alloy or the prosthetic structure made of the alloy is an anticorrosive response to various external stimuli.

Bio-inert metal biomaterials are materials that, once introduced into the human body, have the ability to generate a separation film at the interface with the biological tissue. The objective of the study was to determine the tendency of passivation and stability of the oxide film formed on the surface of the alloy. Only three out of five Co–Cr alloys were suitable as raw materials for the new direct casting method, and the starting hypothesis was that the same alloys have the best response in terms of passive film generation and protection.

#### 3.1.1. Open Circuit Potential (OCP) Measurement

The assessement of the variation of the potential of the alloys as working electrodes as a function of time in open circuit (OCP) conditions provided information on the stability of materials immersed in simulated biological fluid (Ringer’s solution that mimics the salt composition of saliva), but also information on the electrochemical behavior of these materials in terms of electrical conductivity (the conductive or insulating property of the material).

Measurement under open circuit potential conditions showed the tendency of the alloy to create a passive layer on the analyzed surface when in contact with an electrolyte that mimics the composition of saliva. This electrically passive layer (insulator) that had formed on the exposed surface of the alloy was considered an anticorrosive response.

**Figure 2:**
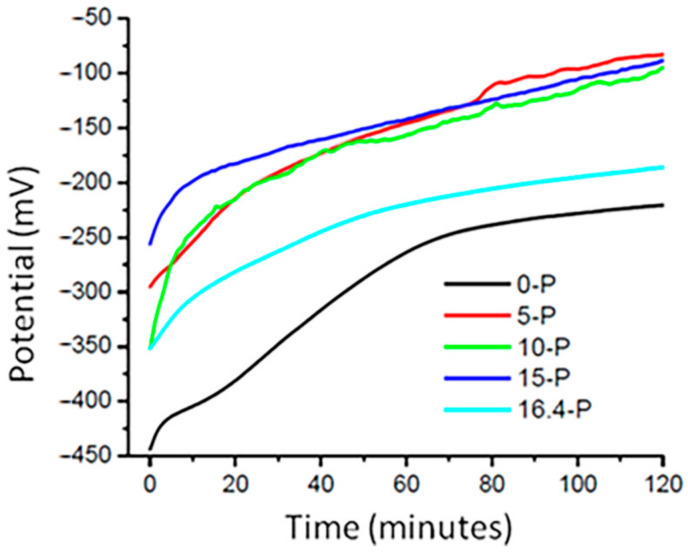
The time variations of the open circuit potentials at the surfaces of the alloys studied in contact with the Ringer’s solution medium.

The time variation of the electric potential at the level of the electrode work (sample area) was measured in relation to the reference electrode and in the presence of the auxiliary electrode, all being immersed in the Ringer’s solution. The duration of the experiments was 120 min, and the result obtained is shown in Figure 2.

The values of the initial potentials and after 120 min in contact with Ringer’s solution that were recorded for the alloy samples are presented comparatively in Table 1. Differences were also calculated potentially appropriate for a critical discussion regarding the properties of tested surfaces. At least three tests were performed for each alloy sample using the mean value of the potential difference, and the standard deviations of these tests are also included in Table 2.

As shown in Figure 2, during the first tens of minutes, on the surface of the working electrode a passive film with the characteristics of corrosion protection was formed, which are expressed in the diagram by the almost constant level (a very small variation of the potential being registered compared to the first minutes of the experiment). In the case of some materials, the trend of stabilizing the potential signal was achieved very quickly, as in the cases of samples 15-P and 16.4-P, whereas for the 0-P sample the tendency for the potential stabilization was slower. For the 15-P and 16.4-P samples, the potential jump from the initial moment until reaching the stabilization plateau was the smallest, suggesting passivation by an oxide coating and, implicitly, anticorrosive resistance. Thus, the general tendency of anodization during the registered potential was observed from the maximum values (−443.62 mV and −256.37 mV) to the minimum values (−220.75 mV and −83.12 mV) with differences of potential (ΔE) in the range from 256.87 mV to 165.2 mV.

Even if the values of the registered potential were different, the potential difference shows the similar tendency of passivation of the tested materials; this aspect is indicated by the curves represented in Figure 2. The tendency for stabilization of the potential shows that the passive film had really formed, and this parameter is graphically represented by the last third at the end of the described curves.

#### 3.1.2. Electrochemical Impedance Spectroscopy (EIS)

EIS was used to characterize the passive layer on the surfaces of the analyzed alloys. EIS data were plotted as a Nyquist diagram, and the parameters of the equivalent electrical circuit were determined to obtain important information regarding the nature and properties of the coatings on the surfaces of the tested alloys.

**Figure 3:**
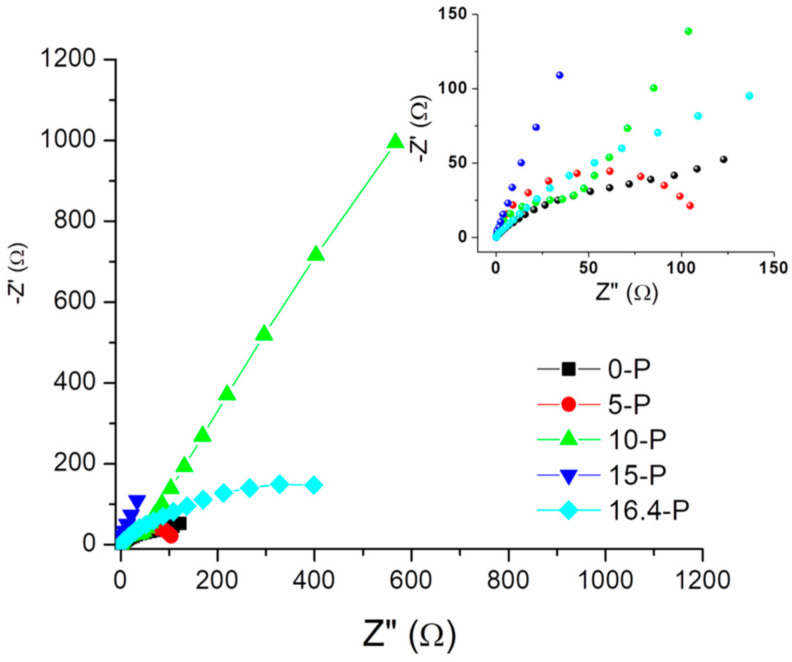
The Nyquist diagram of the EIS for the five alloy samples.

The experimental data of impedance spectroscopy were modeled using a mathematical system called electrical circuit equivalent. It contains various elements, such as resistors, capacitances, inductances, etc., which ensure the characterization of the material electrode and the processes that take place at the electrode interface. EIS tests allowed the determination of the polarization resistance (R_p_) of the alloys after processing by casting. A representation of the EIS in the form of a Nyquist diagram highlights the electrochemical behavior of alloys at polarization (Figure 3). The representation of the recorded values suggest differences among the electrochemical behaviors of the five alloy samples obtained after casting, and therefore the electrochemical behaviors at the surfaces each sample were critically interpreted.

**Figure 4:**
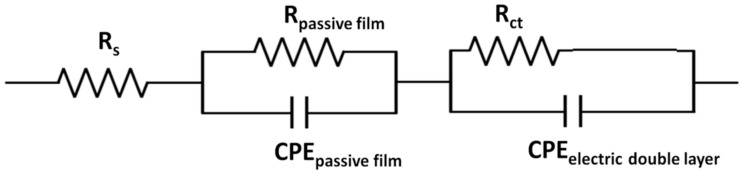
Equivalent Randles-type electrical circuit proposed for modeling processes at the alloy sample-type working electrode interface after casting treatment.

Taking into account the nature of the electrode surface for the alloys tested after casting treatment, it was proposed to model the equivalent electrical circuit, which can be seen in Figure 4. The constituent elements of this circuit were: the resistance of the electrolyte solution (R_S_), the resistance of the pores in the passive film generated at the surface (R_passive film_), the pore capacitance of the passive film, which was expressed here by the constant phase element (CPE_passive film_), the charge transfer resistance (R_ct_), and the capacitance of the electric double layer at the interface of the metallic material (alloy) expressed by the constant phase element (CPE_electric double layer_). All these components of the proposed circuit are illustrated in Figure 4. For a better understanding of the phenomena accompanying the redox process at the working electrode, the values of the equivalent electrical circuit parameters determined for the five tested materials are presented in Table 3. The lowest value of R_ct_ was recorded for sample 15-P, and the highest was recorded for sample 16.4-P.

#### 3.1.3. Linear Polarization Measurement

Linear polarization studies were performed by sweeping the potential in the range from −1000 to +1000 mV.

**Figure 5:**
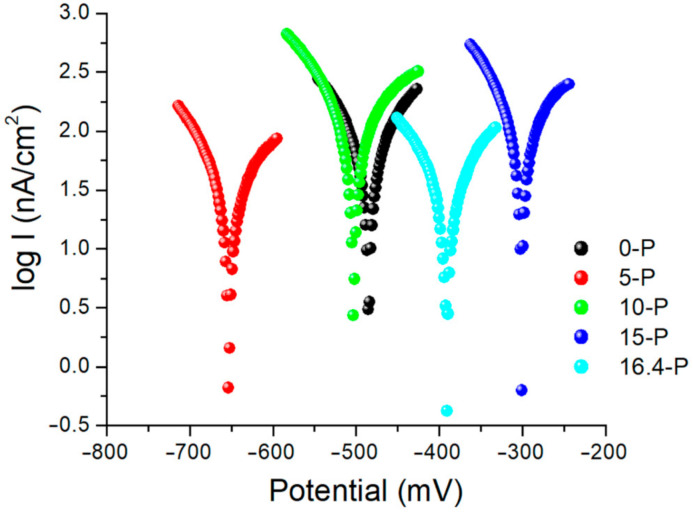
Comparative representation of the potential dynamics curves for the samples 0-P (black); 5-P (red); 10-P (green); 15-P (blue) and 16.4-P (light blue) analyzed after casting. The tests were performed in the presence of Ringer’s solution. The estimation of linear polarization at corrosion can be conducted by the interpretation of Tafel diagrams. Corrosion current density, which is expressed related to the loss in thickness of the material from the surface for one year, was determined from Tafel’s equation graphically represented by Tafel diagrams of linear polarization (data not presented) and with logarithmic values of corrosion current density.

Tafel type graphs with a current density logarithm are shown in Figure 5 for all alloy samples considered. The 15-P alloy had the lowest polarization potential, suggesting the best corrosion resistance, followed by the alloy 16.4-P and the alloys 0-P and 10-P, and the alloy 5-P was the least resistant.

The tests referred to the application of a direct current over a potential range with a speed of 1 mV/s and the recording of the current density that was generated at the analyzed surface. The thermodynamic parameters β_a_ and β_c_ from the Butler–Volmer equation were also determined, and the values obtained for all the mentioned quantities are presented in Table 4.

The 15-P alloy had the lowest negative value of the potential recorded at zero current, suggesting the best anti-corrosion resistance; these results confirm the observations made in previous tests for determining the OCP and electrochemical impedance (Table 3).

#### 3.1.4. Electrochemical Impedance Studies to Characterize the Properties of the 15-P Alloy after Various Processing Steps

Electrochemical impedance spectroscopy was used to characterize the surface of the 15-P alloy after performing some processing procedures. This type of alloy was chosen for further studies based on the results of the comparative studies presented above.

**Figure 6:**
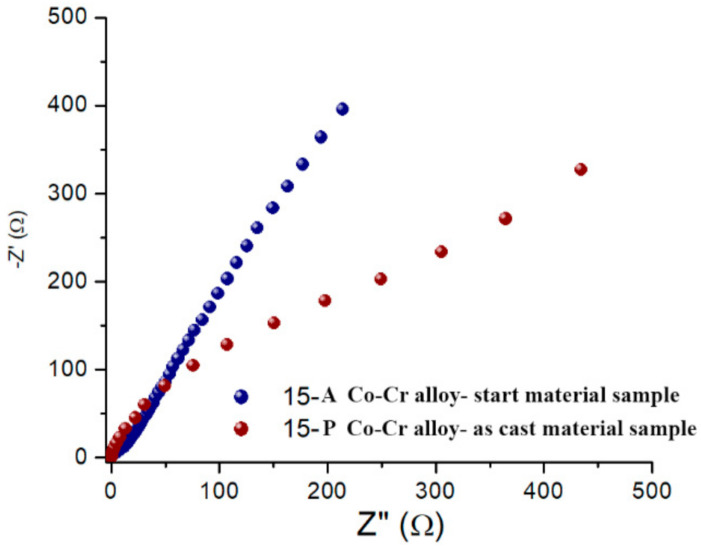
EIS Nyquist diagrams for start material sample 15-A alloy (ingot) (blue) and as cast material sample casting 15-P (brown).

The Nyquist diagrams of the EIS presented in Figure 6 indicate that the casting processing of the 15-S alloy slightly increased the electron transfer resistance from 50 to 100 Ω; therefore, the obtained material had lower conductive properties compared with the starting material. However, the general appearance of the Nyquist representation of EIS did not change, as evidenced by the fact that the alloy did not change its composition after casting.

**Figure 7:**
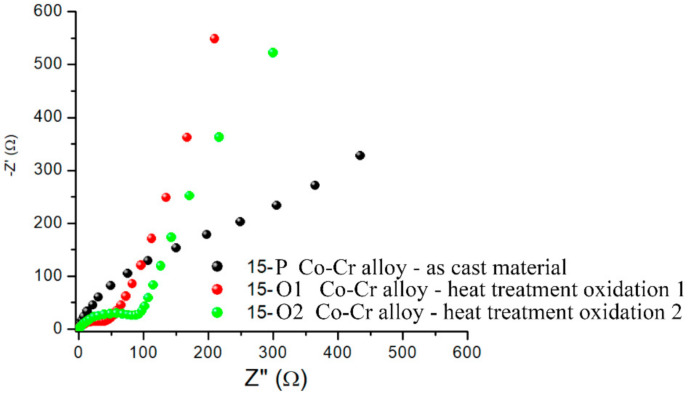
Nyquist representation of EIS for 15-P alloy after casting (black) and after oxidation by heat treatment by Protocol 1 (red) and heat treatment by Protocol 2 (green).

As it can be observed in the Nyquist plots of the EIS (Figure 6), oxidative heat treatment caused improved conductive properties of the 15-P alloy sample surface, whereas the R_ct_ value was lower after the use of Protocol 1 for the oxidation process (see Figure 7: red).

### 3.2. Scanning Electron Microscopy and EDS Spectrometry

SEM offered the possibility to study the surface morphology of the alloy samples 5 (5-P and 5-O3) and 15 (15-P and 15-O3), representing the samples from the molten and cast alloy (P) and the samples after the three cycles of oxidative heat treatment (O3). By SEM, the influence of different factors was assessed, including the composition of the studied samples and the treatments performed on the surface topography.

**Figure 8:**
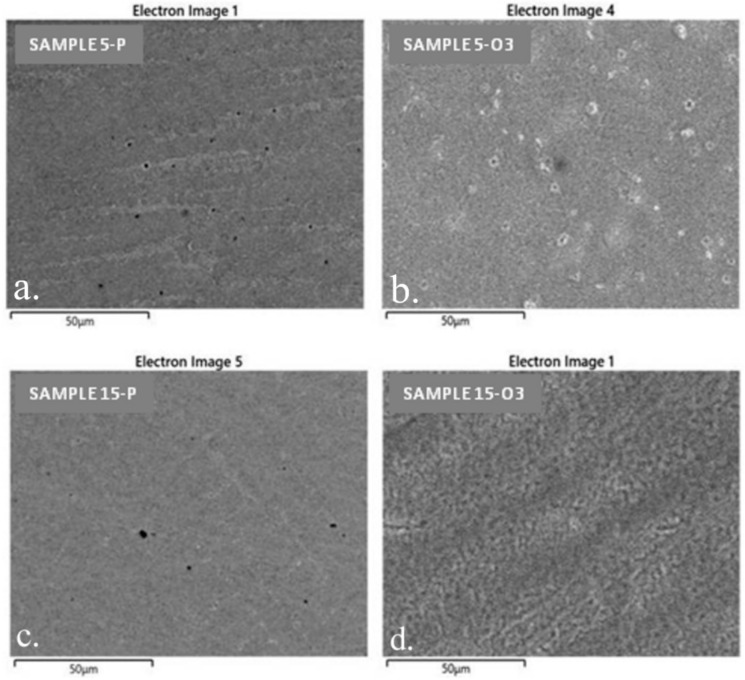
SEM images: (**a**) sample 5-P; (**b**) sample 5-O3; (**c**) sample 15-P; (**d**) sample 15-O3.

The SEM images of the two studied samples after heat treatment, Sample 5 and Sample 15 (Figure 8), highlight the formation of a crystalline layer on their surfaces. EDX analyses revealed that this layer was composed of the two types of oxides specific to Co and Cr. Subsequent X-ray diffraction analyses confirmed these findings.

**Figure 9:**
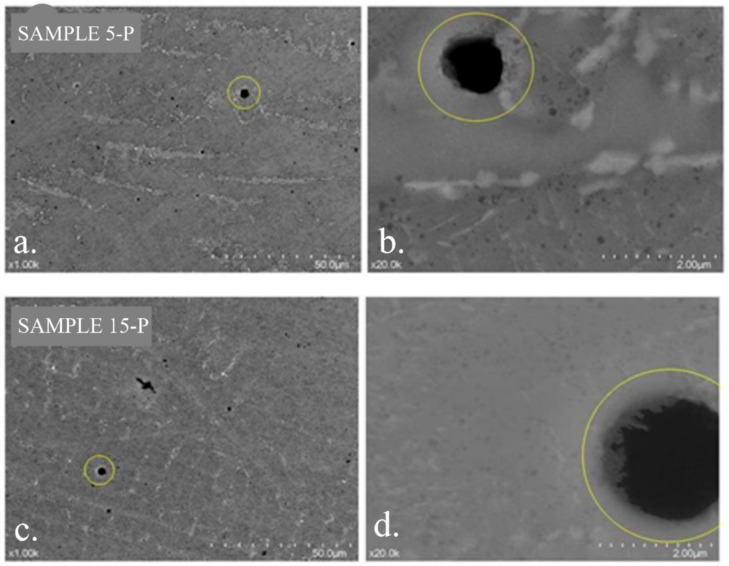
Surface SEM analysis of the samples, demonstrating the presence of pores (marked with a yellow circle): (**a**,**b**) sample 5-P; (**c**,**d**) sample 15-P.

The SEM images of samples 5-P and 15-P show that they had a porous surface. The pore density on the surface of sample 5-P was higher than that on the surface of sample 15-P (Figure 9). On the surfaces, there also were traces remaining after mechanical processing.

**Figure 10:**
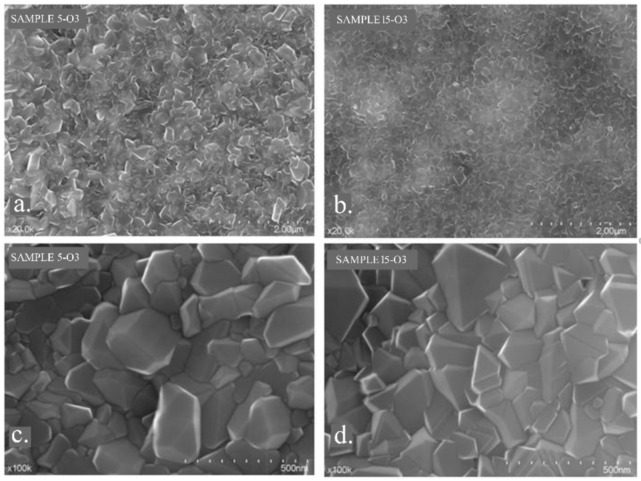
Surface SEM images: (**a**,**c**) sample 5-O3; (**b**,**d**) sample 15-O3.

Surface SEM images obtained under higher magnification show, for sample 5-O3, an inhomogeneous polycrystalline structure and the presence of differently shaped and sized crystals, suggesting an uneven contribution of the two types of oxides. However, similar SEM images of sample 15-O3 show a more homogeneous crystalline structure regarding the crystal size (Figure 10).

**Figure 11:**
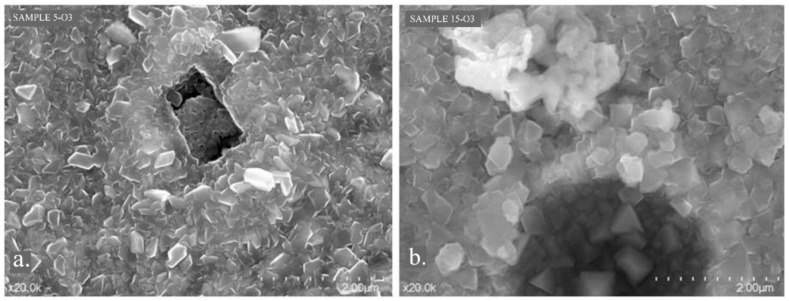
The defects at the surface of the oxide layers: (**a**) sample 5-O3; (**b**) sample 15-O3.

Defects in the oxide layer could be observed on the surfaces of samples 5-O3 and 15-O3, in addition to the pores. The shape and volume of the detected defects differed from sample 5-O3 to sample 15-O3. The defect on the surface of sample 5-O3 was shaped more rectangular, and there was a lack of substance under the surface layer (Figure 11a). In sample 15-O3, the defect had an ovoid shape and a tunneling appearance (Figure 11b).

### 3.3. Energy-Dispersive X-ray Spectroscopy: EDS

EDS was used to analyze the elemental composition of the sample surfaces. X-ray emissions were stimulated by irradiating the surface with an electron beam or a focused X-ray beam.

**Figure 12:**
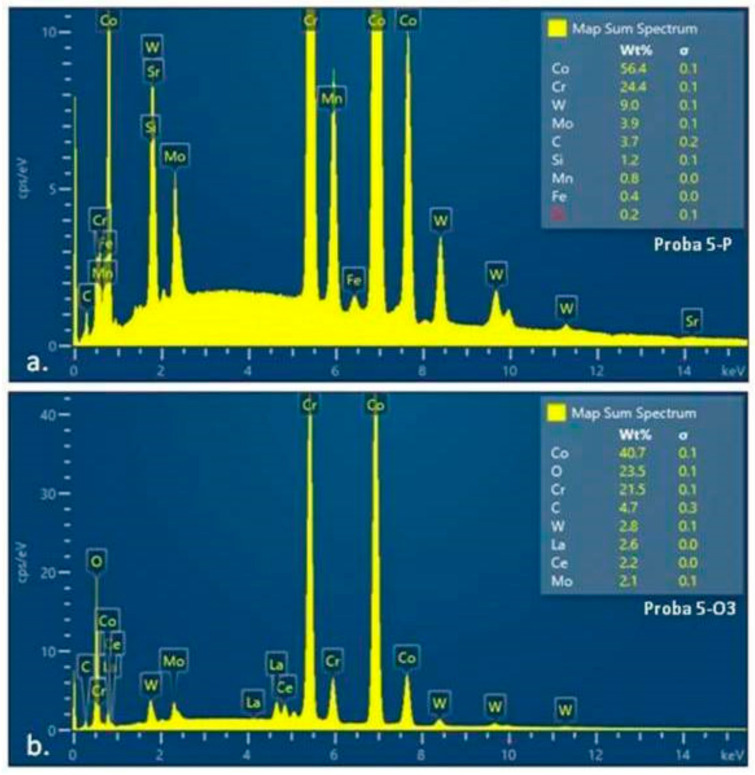
Diagram of EDS spectra with elemental analysis: (**a**) sample 5-P (before heat treatment); (**b**) sample 5-O3 (after heat treatment).

**Figure 13:**
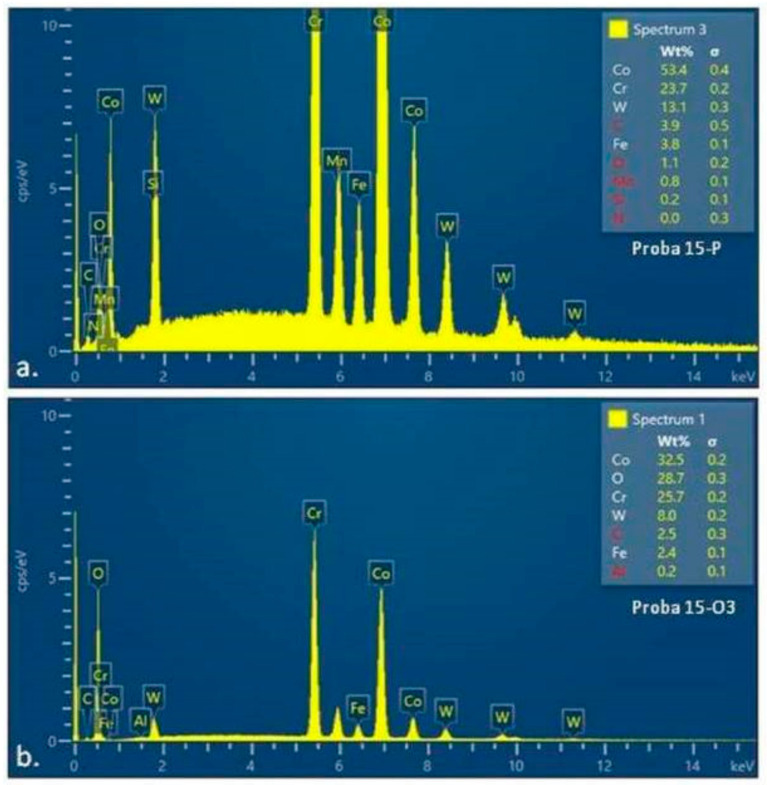
Diagram of EDS spectra with elemental analysis (**a**) sample 15-P (before heat treatment); (**b**) sample 15-O3 (after heat treatment).

The EDS spectra of material sample 5 (5-P and 5-O3) (Figure 12) and material sample 15 (15-P and 15-O3) (Figure 13), analyzed by SEM, allowed the determination of the elemental compositions of the surface layers of the two different Co–Cr-based alloy, which were previously subjected to heat treatment for the surface distribution of the elements.

The values representing the percentage compositions of the elements present on the surface of the examined samples (tables indicated in the EDS spectra of samples 5-O3 and 15-O3) indicate the majority presence of Co, Cr, and O elements, corresponding to the chemical structures of the oxides of the two metals (Table 4 and Table 5). Although the quantitative values of the component elements could not be considered as perfectly relevant, both due to the signal uptake below the oxide surface and due to method errors, it could be concluded that the heat treatment led to the formation of a consistent oxide layer. From the ratio of Co and Cr quantities detected initially and after heat treatment on the two categories of samples, it could be concluded that, in alloy 5 (with 5% W), Co oxidation was more significant (Figure S8 and Table 5), whereas for alloy 15 (with 15% W), Cr oxidation predominated (Figure S9 and Table 6).

The value of 28% oxygen content in sample 15 could correspond to a mixture; on the surface of sample 5, the average value of 15% oxygen suggested either a thinner layer of oxide, or a larger amount of Co oxide, which had a lower proportion of oxygen. Taking into account the value of 72% Co content before and after heat treatment, it could be concluded that the second hypothesis is valid, but the X-ray diffraction analyzes were required for validation. In conclusion, the EDS analysis of samples 5-O3 and 15-O3 confirmed the formation of Co and Cr oxides respectively on their surface due to the heat treatment performed.

### 3.4. X-ray Diffraction

**Figure 14:**
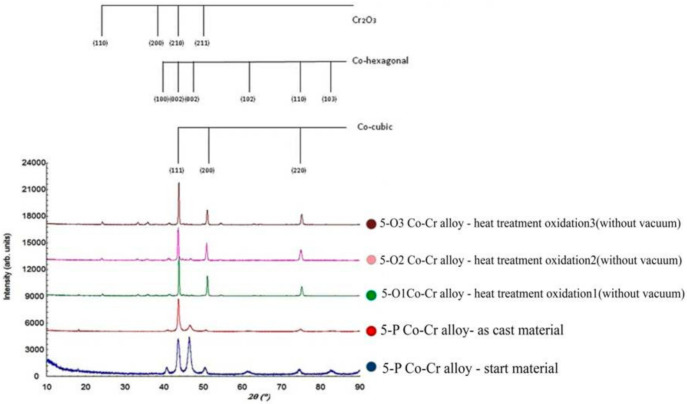
X-ray diffractograms for sample 5.

**Figure 15:**
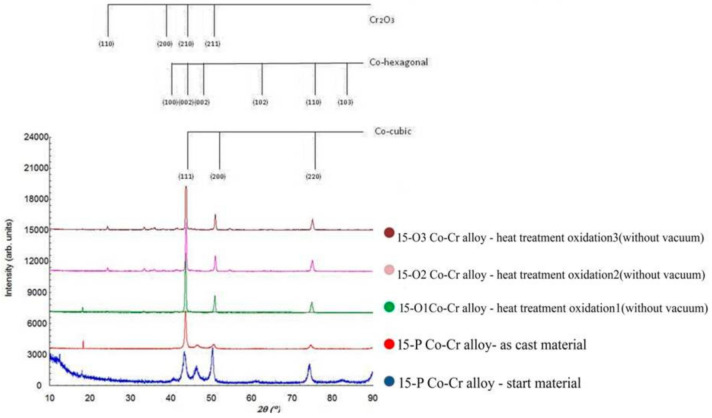
X-ray diffractograms for sample 15.

The surface analysis of the A samples (5-A, 15-A) revealed that the crystal structure was consistent with the structure for cobalt. In the samples, two phases of Co were found: the cubic phase with centered faces and the hexagonal phase with compact faces (Figure 14 and Figure 15).

All the structural elements of the samples formed cubic structures with centered faces and the space group Fm-3m. Thus, the following lattice parameters were obtained:

- Co; a = 3.54 Å;
- Cr; a = 3.6 Å;
- Fe; a = 3.64 Å;
- Mo; a = 4.03 Å;
- W; a = 4.06 Å;

These elements also formed the hexagonal structure for:

- Co; a = b = 2.507 Å, and c = 4.08 Å.

Similar structures formed Cr and Fe, with close lattice parameters.

Therefore, it was expected that Cr, Fe, and possibly W atoms would enter the Co structure. The first three diffraction lines had the following Miller indices: (111), (200), and (220). The maximum diffraction with Miller index (200) was more intense than that given in the database (PDF 2), whereas the maximum diffraction corresponding to Miller index (111) was less intense than in the database (PDF 2). This can be explained by the preferential orientation of the crystallites in such a way that the crystallographic plane (200) was parallel to the plane of the sample.

The positions of the experimental diffraction lines differed from the positions of both of the diffraction lines for Co or Cr and depended on the quantities of each element that had entered the lattice. for both the cubic phase and the hexagonal phase.

By the fact that the lattice constants differed from the lattice constants of the pure elements, and because no crystalline phase other than the cubic and the hexagonal phase has been identified, it resulted that a solid solution was formed for both the cubic and the hexagonal phases. Both elements included several elements.

After processing by melting and casting, the preferential orientations for the two samples (5-P and 15-P) disappeared, and the two cubic and hexagonal phases were maintained.

Next, the samples were oxidized, and the X-ray diffraction patterns for samples 5-O1, 5-O2, and 5-O3 are shown in Figure 14. For samples 15-O1, 15-O2, and 15-O3, the diffraction patterns are similar (Figure 15). The hexagonal phase had completely disappeared, and new phases appeared. The new phase that appeared was of type (A, B)_2_O_3_. This phase had the spatial group R-3c, and there were a series of compounds with network constants close to the values a = b = 5.0 Å and c = 13.7 Å. Among them, we can mention Fe_2_O_3_, Cr_2_O_3_, (Cr, Fe)_2_O_3_, CoMnO_3_, etc.

For samples 5-A and 15-A, both the crystalline phase corresponding to hexagonal Co and the crystalline phase corresponding to cubic Co were obtained. The crystalline phase for hexagonal Co had the spatial group P63 / mmc with the parameters of the elementary cell a = 2.55 Å, c = 4.09 Å, and the crystalline phase corresponding to cubic Co had the spatial group Fm-3m and the parameter of the elementary cell 3.58 Å.

The parameters of the elementary cell were slightly higher because the Co lattice also included Cr and W atoms that had a slightly larger ionic radius.

For the 5-P and 15-P samples, the amount of hexagonal phase decreased slightly, and the cubic phase increased. For the samples to which oxidative heat treatment was applied, the hexagonal phase almost disappeared. Moreover, starting with samples 5-O1 and 15-O1, Cr_2_O_3_ also appeared on the surface, which further developed for samples 5-O2 and 5-O3 as well as 15-O2 and 15-O3.

For the hexagonal and cubic phase of Co, the crystallites siezes were calculated using Scherrer’s relation: D = (0.9λ)/(βcosθ), where D is the crystallites siezes, λ is the X-ray wave length, θ is the diffraction angle, and β is the width at half height of the diffraction line corrected by the instrumental broadening. The results are presented in Table 7.

For samples 5-P and 15-P, the crystallite size slightly decreased compared to samples 5-A and 15-A due to the phase transformation process from the hexagonal phase to the cubic phase, which began to take place in this stage of sample processing. After this, for samples 5-O1 and 15-O1, the crystallite dimensions increased significantly while, for samples 5-O2 and 5-O3 as well as 15-O2 and 15-O3, they tended to remain constant.

## 4. Conclusions

All five alloys showed an anodizing tendency, represented by the maximum negative values that tended towards minimum negative values. A passive layer on the surface of the samples was formed during the first minutes of the application of the electric potential in open circuit conditions, and it was maintained at the surface of the alloy samples as indicated by the stabilization of the registered potential. The lowest negative values were recorded for the same three alloys validated by the direct casting method, namely 5-P, 10-P, and 15-P.

After measuring, linear polarization, and the graphical representations of the values for the three valid alloys, a more pronounced anodizing tendency was observed in the Tafel diagram for the alloy that had a higher percentage of W and Fe in the composition, Co 55.2 Cr 24.0 W 15.0 Fe 4.0 Si 1.0 Mn 0. The highest negative values, representing a weak tendency of anodizing and passivation at the surface, were recorded for the alloy Co 59.5 Cr 31.5 Mo 5.0 Si 2.0 Fe 1.0 Mn 1.0, also invalidated by the direct casting method.

The Nyquist representation of EIS shows that the electrode surface represented by the alloys had undergone changes in roughness or porosity, or it had developed and deposited on the surface corrosion products that could be of the oxide type, thus confirming the above observations.

After applying the heat treatment on the cast alloys, an oxide separation film resulted on the surface samples, and therefore it favored the manufacturing of removable assemblies of prosthetic structures by the method of direct casting.

In the analysis of the surfaces of the samples as start materials, the resulting crystalline structure was consistent with Co in two phases: the cubic phase with centered faces and the compact hexagonal phase. Similar structures formed Cr and Fe with similar lattice parameters, so it was expected that other atoms would enter the Co lattice, the amount of which could influence the position of the experimental diffraction lines. After melting and casting the alloys, the preferential orientations of the crystallites disappeared, but the two cubic and hexagonal phases remained.

After the application of the heat treatment, the hexagonal phase disappeared, and the appearances of new phases of type (A, B)_2_ O_3_ corresponding to some oxide compounds such as Fe_2_O_3_, Cr_2_O_3_, (Cr, Fe)_2_ O_3_, and CoMnO_3_ were observed.

The dimensions of the crystallites decreased slightly after melting and casting the alloys as an effect of the phase transformation process from hexagonal to cubic, and they increased in size after each stage of the heat treatment.

The EDS data revealed the formation, after heat treatment, of a passive layer composed of oxides specific to cobalt and chromium, which is consistent with experimental XRD results and EDS values.

## Figures and Tables

**Table 1:** Chemical composition of the experimental Co–Cr alloys; (x—content ≥ 1%, - chemical element not present) (alloy 0 [35] alloy 5 [36], alloy 10 and alloy 15 [37,38], alloy 16.4 [39]).

Alloys	Co [%]	Cr [%]	Mo [%]	W [%]	Fe [%]	Si [%]	C [%]	Mn [%]	N [%]	Ce [%]	Nb [%]
0	59.5	31.5	5	-	x	2	-	x	-		-
5	61	26	6	5	0.5	1	0.02	-	-	0.5	0.9
10	59	25	4	10	-	1	-	0.8	0.2	-	-
15	55.2	24	-	15	4	1	-	0.8	x	-	-
16.4	54.1	20	-	16.4	7.5	1.5	-	0.3	-	-	0.2

**Table 2:** Values of the open circuit potential over time on the surfaces of the alloy samples.

Alloys	E_0_ (T = 0 min) (mV)	E_120_ (T = 120 min) (mV)	ΔE (mV)	RSD (%)
0-P	−443.62	−220.75	222.87	3.4
5-P	−295.31	−83.12	212.19	2.7
10-P	−351.87	−95.00	256.87	1.9
15-P	−256.37	−88.56	167.81	2.1
16.4-P	−351.50	−186.31	165.20	4.3

**Table 3:** Parameters of equivalent electrical circuits determined for the five tested materials.

Sample	R_S_ [Ωcm^2^]	R_ct_ [Ωcm^2^]	CPE_electric double layer_ [F/cm^2^]	CPE_passive film_ [F/cm^2^]
0-P	16,296	232,660	1.3204 × 10^−5^	0.88785
5-P	15,437	537,080	1.4201 × 10^−5^	0.87963
10-P	16,934	176,901	1.6782 × 10^−5^	0.87001
15-P	14,553	146,120	1.6131 × 10^−5^	0.87704
16.4-P	14,306	509,751	13987 × 10^−5^	0.83287

**Table 4:** Values of E, I_cor_, β_a_, β_b_, V_cor_.

Alloys	E(I = 0) [mV]	I_cor_ [μA/cm^2^]	R_p_ [kohm/cm^2^]	β_a_ [mV]	β_c_ [mV]	V_cor_ [μm/an]
0-P	−484.7	73.2902	243.23	112.4	−103.2	1.0960
5-P	−652.6	35.4831	537.08	140.7	−91.00	0.5308
10-P	−502.9	141.57	178.91	214.1	−118.8	2.1170
15-P	−300.9	86.4078	161.62	113.2	−71.8	1.2920
16.4-P	−391.0	35.7285	511.57	117.2	−106.6	0.5344

**Table 5:** Quantitatively determined values of the content of the elements on the surface of sample 5-O3.

Elements	wt%	wt% Sigma
C	4.74	0.26
O	23.46	0.13
Cr	21.48	0.08
Co	40.75	0.14
Mo	2.06	0.06
La	2.58	0.04
Ce	2.16	0.04
W	2.78	0.06
Total:	100.00	

**Table 6:** Quantitatively determined values of the elements at the surface of sample 15-O3.

Elements	wt%	wt% Sigma
C	2.70	0.15
O	27.36	0.12
S	0.02	0.02
Cr	19.50	0.07
Fe	2.84	0.03
Co	38.38	0.11
W	9.19	0.09
Total:	100.00	

**Table 7:** Crystallite dimensions for the crystalline phases of Co.

Sample 5	5-A	5-P	5-O1	5-O2	5-O3
Crystallite size hexagonal Co (Å)	153	128	-	-	-
Crystallite size cubic Co (Å)	287	266	456	500	556
Sample 15	15-A	15-P	15-O1	15-O2	15-O3
Crystallite size hexagonal Co (Å)	142	114	-	-	-
Crystallite size cubic Co (Å)	210	190	406	412	442

## Data Availability

Not applicable.

## Supplementary Materials

The following supporting information can be downloaded at: https://www.mdpi.com/article/10.3390/ma15093052/s1. Figure S1: (a,b) Sectioning the Cobalt-Chrome alloy cylinders in horizontal plane, Figure S2: Mechanical processing; (a) abrasive tools; (b) rubber; (c) fibers (cotton wool), Figure S3: Duplicating silicone mold: (a,b) The shape of sample A transposed into the silicone material; (c,d) Fabrication of the born-out model of sample P, Figure S4: (a) Refractory mould inside the preheating furnace; (b) Extraction of the refractory mould from the preheating furnace; (c) Positioning the mold in the melting-casting machine; d. Melting the Co-Cr alloy in a ceramic crucible, Figure S5: TTO parameters on the heating furnace interface: (a) Heat treatment parameters for OTI1 and OTI2; (b) Heat treatment parameters for OC3, Figure S6: (a) Working electrode coupled to the electrochemical cell containing the electrolyte; (b) The electrolyte in the enclosure in contact with the working electrode and the connection of the working electrode to the potentiostat through a crocodile type connector, Figure S7: The location of the reference electrode and the counter electrode on the dedicated stand, Figure S8: Elemental distribution on the analyzed surface EDS for sample 5-O3: (a) the overview; (b) Oxygen (O); (c) Chromium (Cr); (d) Molybdenum (Mo); (e) Cobalt (Co); (f) Wolfram (W), Figure S9: Elemental distribution on the EDS analyzed surface for sample 15-O3; (a) overview; (b) Oxygen (O); (c) Chromium (Cr); (d) Iron (Fe); (e) Cobalt (Co); (f) Wolfram (W).
