# Supplementary material for: Study on the Surface of Cobalt-Chromium Dental Alloys and Their Behavior in Oral Cavity as Cast Materials
Source: Materials (Basel). 2022 Apr 22;15(9):3052. doi: 10.3390/ma15093052 (PMC9101255; doi:10.3390/ma15093052)
Supplement: Supplementary file 1 [file materials-15-03052-s001.zip › materials-1511959-supplementary.pdf]

materials

MDPI

Supplementary Material

Study on the Surface of Cobalt–Chromium Dental Alloys and Their Behavior in Oral Cavity as Cast Materials

The additional information is from the alloy samples process of fabrication: Figures S1 and S2 cutting the start material and surface finishing method.

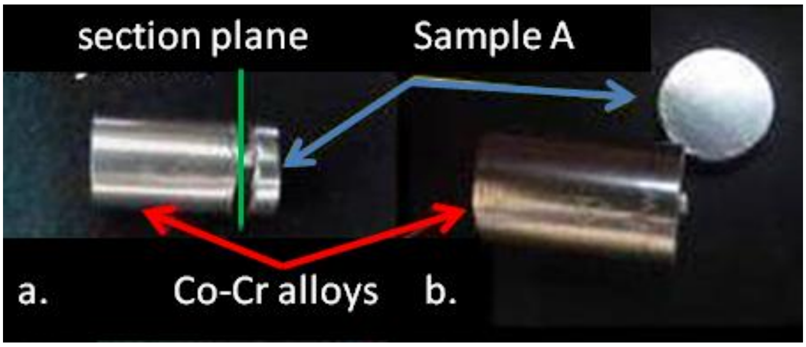

Figure S1. (**a**,**b**) Sectioning the Cobalt-Chrome alloy cylinders in horizontal plane.

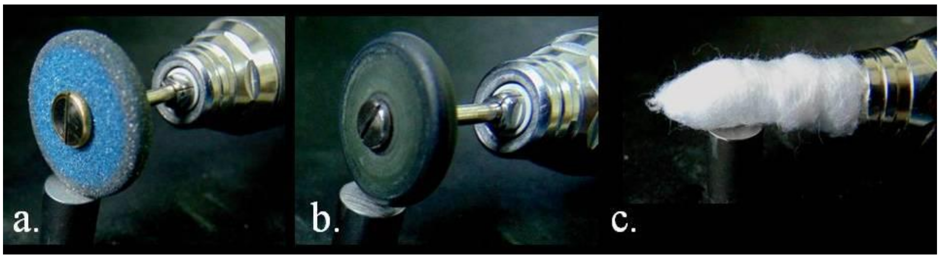

Figure S2. Mechanical processing; (**a**) abrasive tools; (**b**) rubber; (**c**) fibers (cotton wool).

Figure S2 and S3 present the method of fabrication for Samples P- cast alloy.

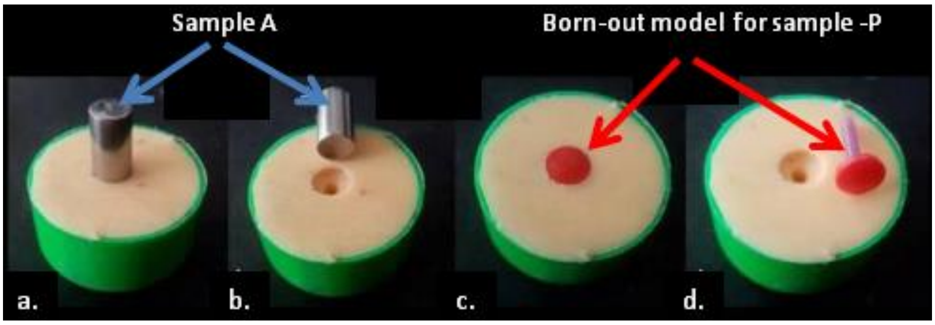

Figure S3. Duplicating silicone mold: (**a**,**b**) The shape of sample A transposed into the silicone material; (**c**,**d**) Fabrication of the born-out model of sample P.

Citation: Uriciuc, W.A.; Boșca, A.B.; Băbtan, A.-M.; Vermeșan, H.; Cristea, C.; Tertiș, M.; Pășcuță, P.; Borodi, G.; Suciu, M.; Barbu-Tudoran, L.; et al. Study on the Surface of Cobalt-Chromium Dental Alloys and Their Behavior in Oral Cavity as Cast Materials. *Materials* **2022**, *15*, 3052. https://doi.org/10.3390/ma15093052

Academic Editor: Iulian Vasile Antoniac

Received: 30 November 2021
Accepted: 21 March 2022
Published: 22 April 2022

Publisher's Note: MDPI stays neutral with regard to jurisdictional claims in published maps and institutional affiliations.

Copyright: © 2022 by the authors. Licensee MDPI, Basel, Switzerland. This article is an open access article distributed under the terms and conditions of the Creative Commons Attribution (CC BY) license (https://creativecommons.org/licenses/by/4.0/).

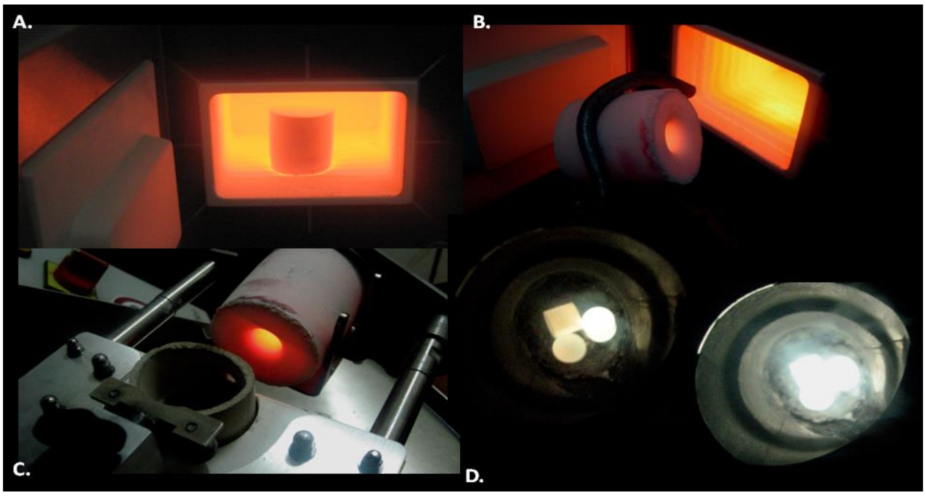

Figure S4. (**a**) Refractory mould inside the preheating furnace; (**b**) Extraction of the refractory mould from the preheating furnace; (**c**) Positioning the mold in the melting-casting machine; d. Melting the Co-Cr alloy in a ceramic crucible.

In Figure S5 is present the set up program for heat treated surface of Samples P:

The oven can be programmed with the following operating parameters (Figure S5):
-B: starting temperature inside the oven (500°C);
-S: time when the oven lid closes (2 min.);
-t: speed of temperature increase (50°C / min);
-T: final temperature (980°C);
-H: time to maintain the enclosure at the final temperature (1min.);
-V1: temperature at which the vacuum pump is activated (500°C);
-V2: temperature at which the vacuum pump is deactivated (979°C);
-L: temperature up to which the enclosure can be cooled slowly.

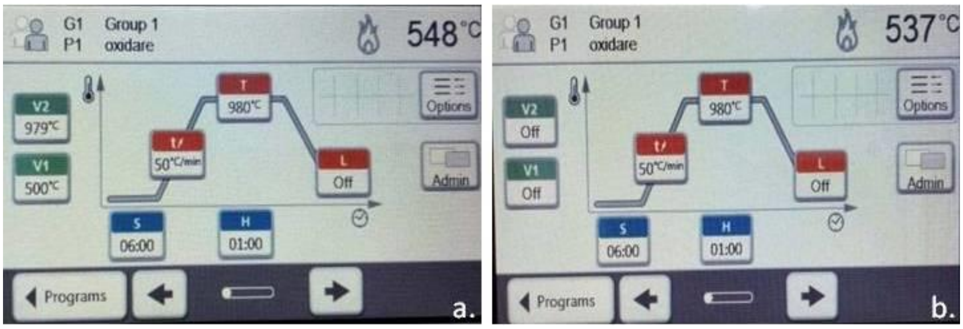

Figure S5. TTO parameters on the heating furnace interface: (**a**) Heat treatment parameters for OTI1 and OTI2; (**b**) Heat treatment parameters for OC3.

Figure S6 and S7 show the electrochemical cell that was develop by the author and the sample insertion.

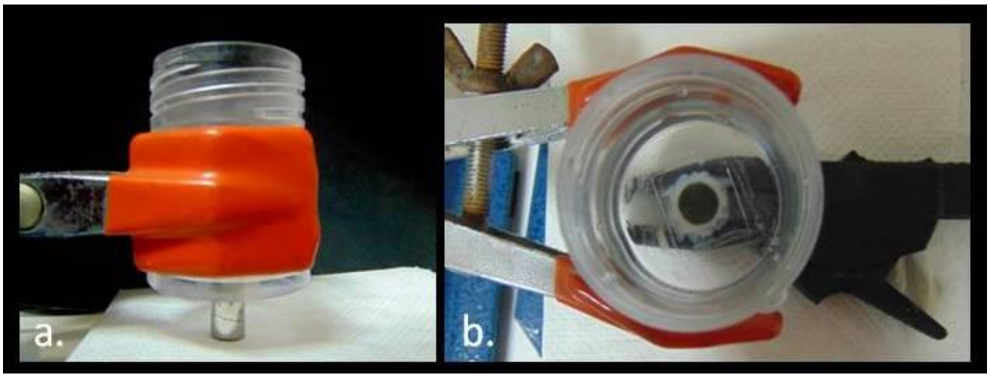

Figure S6. (**a**) Working electrode coupled to the electrochemical cell containing the electrolyte; (**b**) The electrolyte in the enclosure in contact with the working electrode and the connection of the working electrode to the potentiostat through a crocodile type connector.

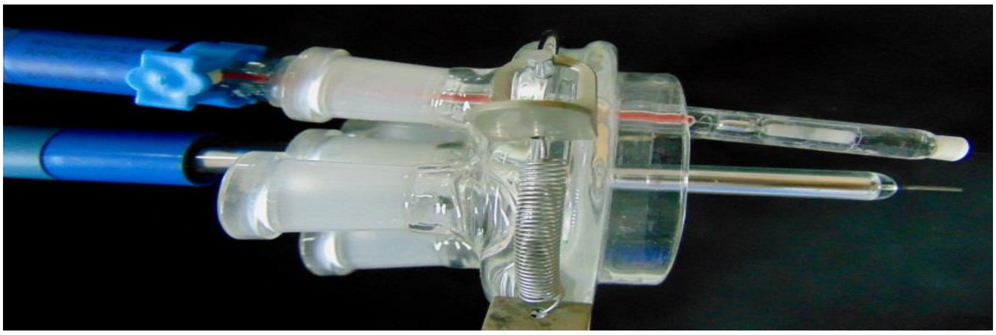

Figure S7. The location of the reference electrode and the counter electrode on the dedicated stand.

Figure S8 and S9 show the elemental distribution on the EDS analyzed surface for samples

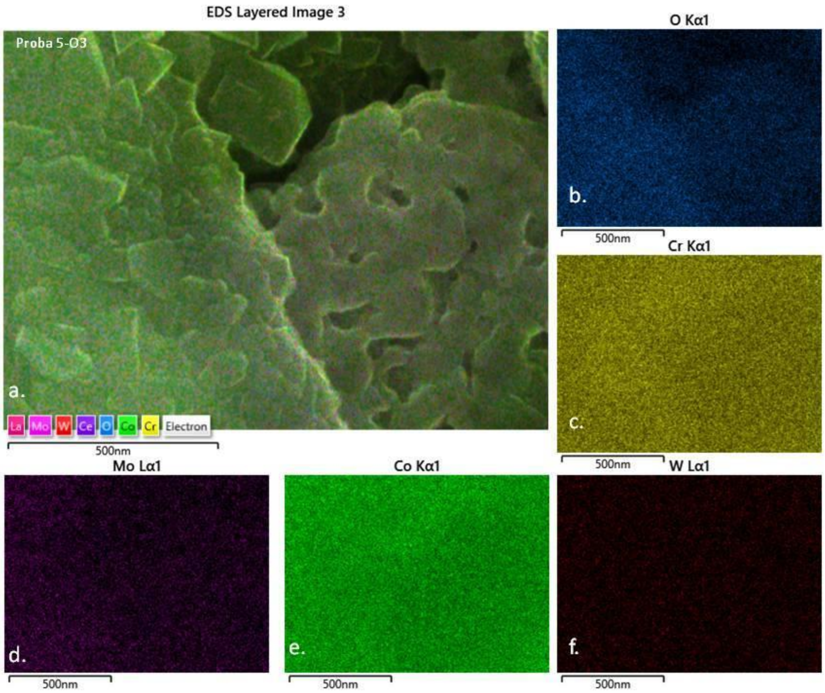

Figure S8. Elemental distribution on the analyzed surface EDS for sample 5-O3: (**a**) the overview; (**b**) Oxygen (O); (**c**) Chromium (Cr); (**d**) Molybdenum (Mo); (**e**) Cobalt (Co); (**f**) Wolfram (W).

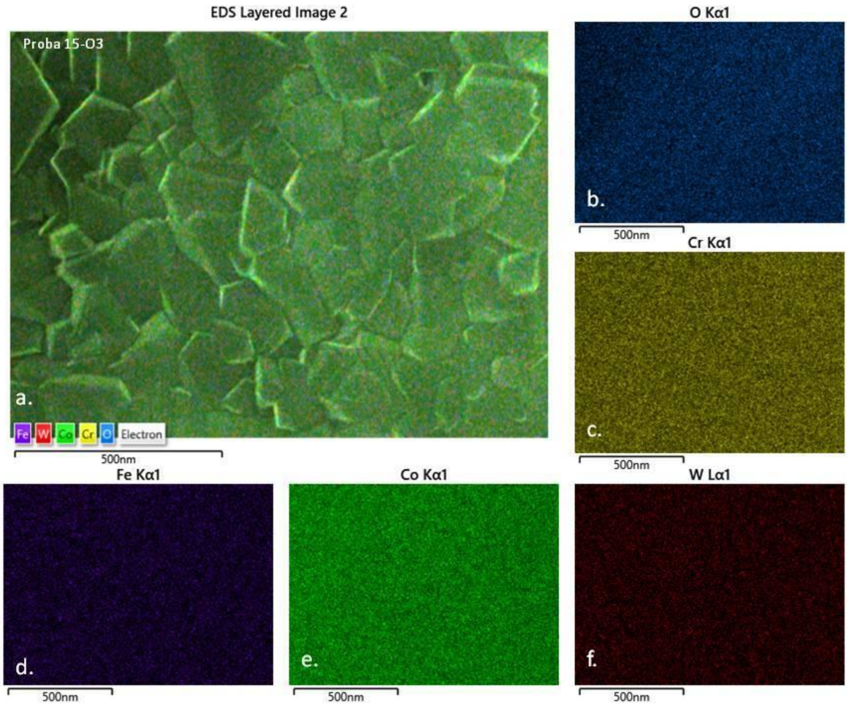

Figure S9. Elemental distribution on the EDS analyzed surface for sample 15-O3; (**a**) overview; (**b**) Oxygen (O); (**c**) Chromium (Cr); (**d**) Iron (Fe); (**e**) Cobalt (Co); (**f**) Wolfram (W).
